# Harnessing Vaccines in the Treatment of Solid Tumors: Advances, Challenges, and Future Directions

**DOI:** 10.3390/vaccines14020135

**Published:** 2026-01-29

**Authors:** Jorge Iranzo, Edoardo Giordano, Renato Maria Marsicano, Dario Trapani, Antonio Marra, Carmen Belli, Paola Zagami, Pier Paolo Maria Berton Giachetti, Emanuela Ferraro, Ida Minchella, Edoardo Crimini, Giuseppe Curigliano

**Affiliations:** 1 Medical Oncology Hospital Arnau de Vilanova, 46015 Valencia, Spain; 2 Division of New Drugs and Early Drug Development for Innovative Therapies, European Institute of Oncology IRCCS, 20141 Milan, Italy; 3 Department of Oncology and Hemato-Oncology, University of Milan, 20122 Milan, Italy

**Keywords:** cancer vaccines, immunotherapy, neoantigens, peptide-based vaccines, nucleic acid-based vaccines, cell-based vaccines

## Abstract

Immunotherapy has become a cornerstone of cancer treatment in both the early and advanced setting in recent years, leading to the achievement of substantial and durable responses with an excellent safety profile across different tumor types. This demonstrates the high potential of engaging the immune system in the treatment of solid tumors. Consequently, there has been renewed interest in vaccines to enhance therapeutic effects, prevent tumor development, and eliminate or control minimal residual disease. Although therapeutic cancer vaccines have shown potential benefits in certain settings, their results in clinical trials remain highly variable and generally unsatisfactory, depending on tumor site, biology, and vaccine type. Currently, Sipuleucel-T for prostate cancer is the only cell-based vaccine that received FDA approval for the treatment of a solid tumor. Innovative techniques such as personalized neoantigen vaccines and mRNA-based vaccines have shown promising preclinical and early-phase clinical results, supporting their further development. Despite the current evidence of vaccine efficacy in treating solid tumors being derived from only a few clinical trials with relatively small sample sizes, ongoing trials are also exploring innovative approaches aimed at preventing cancer development or enhancing immune responses in combination with other immunotherapeutic agents. In this review, we provide an overview of the clinical results and the current state of vaccine development for cancer treatment, outlining future perspectives on their role in managing patients with cancer.

## 1. Background

Cancer remains the second leading cause of mortality worldwide [1]. Significant progress has been made in treating advanced solid tumors with innovative approaches, among which is immunotherapy, which led to improved outcomes in multiple cancer types. Cancer immunoediting [2], a dynamic process divided into an elimination phase, equilibrium phase, and escape phase, governs the interaction between the immune system and tumor cells. During the elimination phase, the immune system tries to eliminate emerging tumor cells. In the equilibrium phase, immune pressure restrains but does not eradicate tumor subclones. Eventually, certain tumor variants bypass immune surveillance, leading to progression.

**Figure 1:**
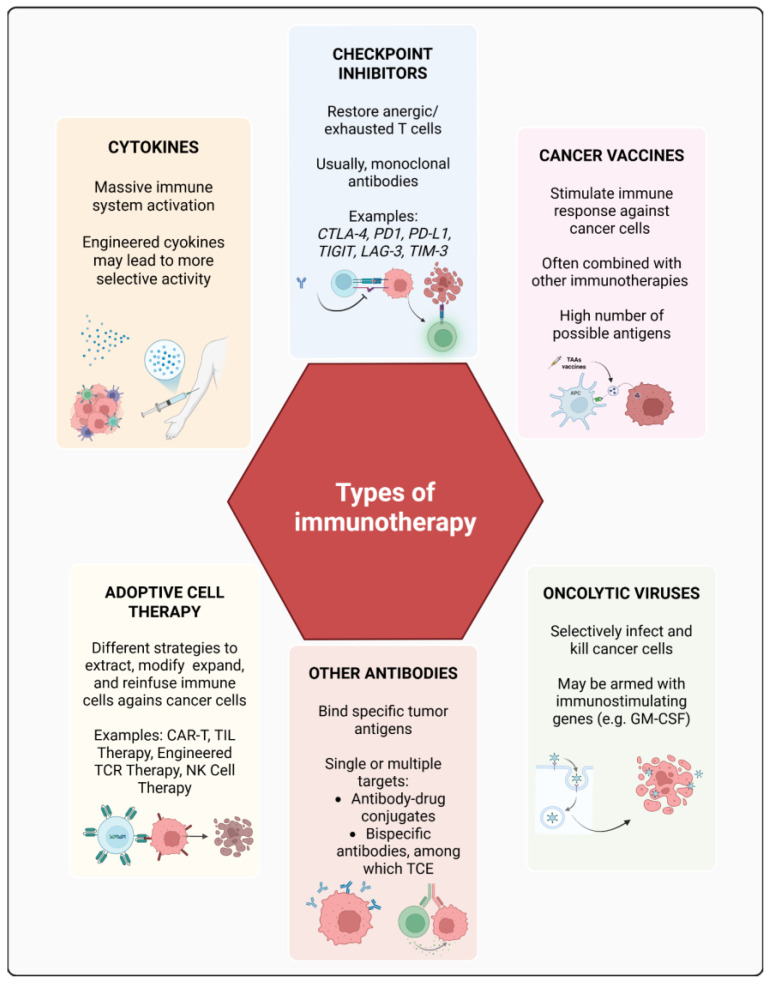
Summary of the main types of immunotherapies. CTLA-4: cytotoxic T lymphocyte-associated protein 4; PD1: programmed death 1; PD-L1: programmed death ligand 1; TIGIT: T cell immunoreceptor with Ig and ITIM domains; LAG-3: lymphocyte activation gene-3; TIM-3: T cell immunoglobulin and mucin-domain containing-3; GM-CSF: Granulocyte-Macrophage Colony-Stimulating Factor. Apart from CPIs and some other exceptions, most mAbs are often referred to as “targeted therapy” even though they also have a direct immunological effect (e.g., trastuzumab binds HER2-expressing cells, activating immune-mediated cancer cell killing).

Immunotherapy can be classified into two approaches: passive and active. Passive immunotherapy uses preformed, immune components like monoclonal antibodies (such as trastuzumab [3] or zolbetuximab [4]) to target tumor-associated antigens (TAAs) [5]. In active immunotherapy, the immune system is stimulated to target and destroy tumor cells. For instance, immune checkpoint inhibitors (ICI) (e.g., anti-PD-1, anti-CTLA-4) activate T cells to enhance antitumor responses, and vaccines train the immune system to recognize and eliminate tumor cells. A summary of the main immunotherapy approaches and mechanisms is shown in Figure 1.

Novel immunotherapies that stimulate both innate and adaptive immunity are being developed to overcome the phenomenon of immune resistance [6]. The ability of cancer vaccines to selectively target cancer antigens and promote tumor elimination through the “cancer-immunity cycle” makes them especially promising among these. Identifying the right target antigens is a significant challenge that is made more difficult by tumor heterogeneity [7] and clonal [8] evolution, which cause dynamic shifts in antigen expression over time. Additionally, vaccines must overcome immune tolerance mechanisms established by the tumor’s microenvironment and various forms of immune resistance [9] that hinder effective immune surveillance [10]. Recent progress in high-throughput analysis allowed the identification of tumor-specific neoantigens as highly promising targets for personalized vaccines. This typically involves whole-exome sequencing to detect mutations unique to malignant cells [11]. Importantly, an effective vaccination needs to induce activation and priming of naïve antigen-specific T cells by professional antigen-presenting cells (APCs), particularly dendritic cells (DCs) [12]. This intricate crosstalk between different immune compartments reflects the difficulties in achieving a strong and durable antitumor response.

Tumor antigens are broadly categorized into TAAs and tumor-specific antigens (TSAs) [13]:

- TAAs are usually expressed in both normal and malignant tissues but are up-regulated in cancer cells, as happens for Human Epidermal Growth Factor Receptor 2 (HER2) and prostate-specific antigen (PSA). Due to their self-origin, TAAs exhibit limited immunogenicity, as T cells recognizing these antigens are unresponsive (anergy) or exhausted during central tolerance. This presents a challenge for vaccine development, which must overcome immune tolerance while maintaining specificity [13].
- TSAs are exclusively expressed by tumor cells and include oncoviral antigens (e.g., Human Papillomavirus-derived) and neoantigens arising from tumor-specific somatic mutations. As they are not subject to central tolerance and are absent from normal tissues, TSAs offer superior immunogenicity and tumor specificity. A higher neo-antigen load has been associated with stronger T cell responses and improved clinical outcomes [13].

Vaccines have been tested widely in many different solid tumors with varying results. The individual characteristics, behavior, current prognosis, and treatment of each tumor make the investigation on therapeutic vaccines different, with different approaches and efforts [14,15,16,17,18,19,20,21,22,23,24,25,26,27,28,29,30,31,32,33,34]. The objective of this review is not to provide an exhaustive catalog of ongoing studies but rather to serve as a concise guide to the fundamental principles underlying the development and application of vaccines in solid tumors, a summary of key vaccine types, their clinical contexts, and ongoing trials, offering an overview of current research efforts.

For completeness’s sake, some vaccines against infectious diseases such as human papillomavirus (HPV) and hepatitis B virus (HBV) play an established and pivotal role in preventing specific types of cancer, but they are out of the scope of this review and will not be discussed.

## 2. Types of Vaccines

**Figure 2:**
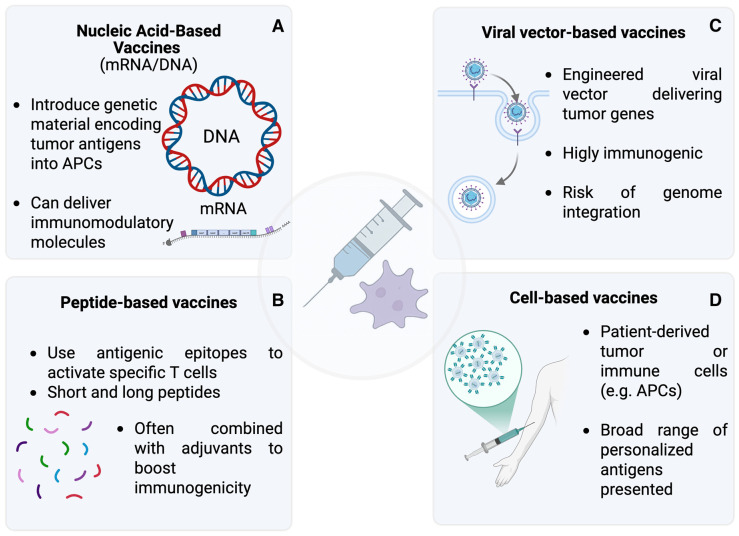
Schematic representation of the four principal classes of therapeutic cancer vaccines: (**A**) nucleic acid-based vaccines; (**B**) peptide-based vaccines; (**C**) viral vector-based vaccines; (**D**) cell-based vaccines.

Cancer vaccines can be broadly classified into four main types, each employing distinct strategies to induce antitumor immune responses [14,15,16,17,18,19,20,21,22,23,24,25,26,27,28,29,30,31,32,33,34] (Figure 2).

### 2.1. Nucleic Acid-Based Vaccines

Nucleic acid–based vaccines represent a promising approach in cancer immunotherapy. These vaccines allow multiple TAAs and TSAs to be delivered into host cells, where translation and antigen presentation further stimulate humoral and cellular immune responses [14].

#### 2.1.1. mRNA

mRNA vaccines can broadly be divided into two categories [15]: conventional (non-replicating) mRNA, which is rapidly degraded after delivery, and self-amplifying mRNA (SAM [16]), which encodes a viral replication machinery to extend antigen expression and increase vaccine immunogenicity [17].

In addition to encoding tumor antigens, mRNA can also be modified to express molecules with immunomodulatory functions, such as cytokines or co-stimulatory signals, that can help to potentiate the immune response. Efficient delivery into the cell is necessary for efficacy, and several approaches to increase uptake and stability have been tried, including naked mRNA, dendritic cell-derived delivery, and protamine-based formulations.

In clinical settings, mRNA cancer vaccines have exhibited limited effectiveness so far [18]. Although their immunogenicity remains restricted and delivery challenges persist, mRNA vaccines have a good profile of safety, high versatility, and rapid and cost-effective production [16]. These features continue to drive their development despite current limitations.

#### 2.1.2. DNA

DNA cancer vaccines are made of closed circular DNA plasmids, often of bacterial origin [19], to encode tumor antigens. After APC internalization, the plasmid DNA enters the nucleus, where it is transcribed into mRNA and then translated into proteins. These proteins act as antigens that are processed and presented to the immune system, eliciting a humoral and cellular response [20].

This xenogeneic bacterial DNA is recognized as foreign, and this activates toll-like receptors (TLRs), inducing TLR9, acting as a danger signal, and cytosolic DNA sensors, inducing a pro-inflammatory response that can help overcome immune tolerance to self-derived TAAs. This innate immune activation enhances the overall immunogenicity of DNA vaccines.

Compared to mRNA vaccines, DNA vaccines are more stable, and they carry a lower risk of degradation. However, they present greater delivery challenges due to their larger size, the need for nuclear localization, and generally require higher doses to be effective. One advantage is that they avoid integrating into the host genome [21], limiting the theoretical oncogenic risk. These limitations, along with their lower immunogenicity, currently make mRNA-based vaccines a more favorable platform for cancer immunotherapy.

### 2.2. Peptide-Based Vaccines

Peptide-based cancer vaccines use highly immunogenic polypeptides containing tumor antigen epitopes. Their effectiveness depends on peptide size, structure, and MHC binding, often being HLA-restricted [22]. Short peptides bind MHC I directly but may be degraded quickly and may induce weak responses. Conversely, long peptides require APC processing, but they may be responsible for a stronger and broader immunity [23].

Adjuvant molecules are commonly used to improve efficacy by enhancing T-cell activation and antigen presentation [24], for example Heat shock proteins (HSP) boost CD8^+^ responses [25], or albumin conjugation improves peptide delivery [26].

These vaccines are safe, easy to produce, and offer precise immune targeting by focusing on key tumor epitopes.

### 2.3. Viral Vector-Based Cancer Vaccines

Viral vector-based vaccines are made of genetically engineered viruses (e.g., adenovirus, herpes simplex virus, lentivirus) in order to deliver tumor antigens and stimulate a strong CD4^+^ and CD8^+^ T cell response [27]. These vectors infect cells, including APCs [28], inducing an immune activation. Different viruses have unique characteristics, which determine vaccine efficacy and limitations. For an in-depth discussion of specific viral vector types, we refer to the relevant literature [29] as this topic falls beyond the scope of this review.

These vaccines typically create a pro-inflammatory environment [30] and may have oncolytic properties, selectively targeting and lysing tumor cells. For example, herpes simplex virus (HSV) targets neuronal cells, making it suitable for treating gliomas [31].

Challenges include the risk of genome integration [32] and the development of anti-vector immunity, which may reduce effectiveness in subsequent doses.

### 2.4. Cell-Based Cancer Vaccines

Cell-based vaccines exploit either immune cells or tumor cells to elicit antitumor immune responses and are broadly classified into immune cell-based and tumor cell-based vaccines.

Immune cell-based vaccines mainly rely on dendritic cells (DCs), which possess a unique capacity to capture, process, and present tumor antigens, thereby initiating antigen-specific T cell responses [33]. In most platforms, immature DCs are generated ex vivo from peripheral blood monocytes, loaded with tumor antigens, and matured using cytokines before reinfusion into the patient [34]. However, the clinical efficacy of DC vaccines has been limited by suboptimal migration to lymph nodes and insufficient in vivo persistence, prompting the exploration of alternative delivery strategies, including in situ vaccination approaches.

Tumor cell-based vaccines can be further subdivided into autologous and allogeneic platforms. Autologous tumor cell vaccines utilize tumor cells or lysates derived from the patient’s own tumor, which are inactivated (typically by irradiation) and often combined with adjuvants or used to load autologous dendritic cells [35,36,37,38]. These vaccines present a comprehensive repertoire of tumor-associated antigens, including patient-specific neoantigens, eliminating the need for prior antigen identification [35,39]. However, autologous vaccines face significant manufacturing challenges, including difficulty obtaining sufficient tumor tissue, labor-intensive production processes, and limited scalability [37,40].

Allogeneic tumor cell vaccines employ tumor cells or lysates from established tumor cell lines or HLA-disparate donors, providing off-the-shelf availability and standardized production [37,38,40]. These vaccines are based on shared tumor-associated antigens expressed across patients with the same cancer type [37,38]. Allogeneic preparations overcome the logistical constraints of autologous vaccines and can be manufactured in quantities sufficient for large-scale trials [39]. Both autologous and allogeneic platforms can be genetically modified to secrete immunostimulatory cytokines such as GM-CSF to enhance antigen presentation and T-cell activation [38,41].

## 3. Vaccines in Different Tumor Types

In this section, we summarize clinical trials with available results in solid tumors in which therapeutic vaccines have demonstrated promising activity. Tumor types were selected based on the availability and maturity of clinical trial data, the number of ongoing or completed vaccine-based studies, and the presence of preliminary signals of clinical or immunological efficacy, to focus on disease settings of greatest current relevance to the field. Key biological and clinical challenges influencing vaccine efficacy and safety, including immune tolerance, tumor heterogeneity, and characteristics of the tumor microenvironment, are discussed within individual trials and tumor-specific subsections.

### 3.1. Melanoma

The introduction of ICIs revolutionized the outcome of patients with melanoma from early to metastatic disease. Consequently, after Ipilimumab approval in 2011, the focus of many researchers shifted from vaccines to this promising novel category of immunotherapy. However, about 50% of patients are resistant to ICIs [42]. Therefore, the combination of ICIs and vaccines could play a role in overcoming the resistance mechanism. In Table 1, we reported ongoing phase II and III trials with vaccine therapy in melanoma patients.

Gp100 was the first TAA discovered in melanoma, and it soon became an appealing target for developing tumor vaccines. In a phase III clinical trial, -gp100 peptide, HLA*A02:01-restricted vaccine was compared with Interleukin-2 alone in patients with advanced melanoma, showing an improvement in overall clinical response (16% vs. 6%, *p* = 0.03), progression-free survival (PFS) from 1.6 to 2.2 months and overall survival (OS)—with a gain of median OS of +6.7 months—in the vaccine plus IL-2 group vs. IL-2 alone [43]. This was one of the first phase III trials that showed benefit from a combination of vaccination and other types of immunotherapies.

NEO-PV-01 is a patient-personalized peptide vaccine, consisting of up to 20 synthetic neoantigen-targeting peptides identified via a proprietary bioinformatics engine (RECON^®^) to select the most immunogenic epitopes in that tumor, studied in a phase Ib trial by Ott et al. in combination with anti-PD-1 therapy in patients with advanced melanoma, non-small cell lung cancer (NSCLC), or bladder cancer. In the vaccinated patients, median ORR (95% confidence interval [CI]) was 59%, mPFS (95% CI) was 23.5 months, and the mOS was not reached in the melanoma cohort. Of note, this study showed a correlation between epitope spread and PFS in patients across all three tumors, meaning that NEO-PV-01 can induce T cells capable of killing tumor cells. Furthermore, from a small subset of neoantigens, it is possible to trigger a wider immune response against more neoantigens not included in the vaccine [44]. However, the single-arm design of the trial is an impactful limitation. More trials, including a control arm with anti-PD-1 monotherapy, are needed to understand whether the tumor regression observed post-vaccination is an effect of the vaccine itself rather than a delayed response to nivolumab.

The mRNA-4157 (V940) is a novel mRNA-based individualized therapeutic vaccine, designed to encode over 30 neoantigen sequences from patient-specific somatic mutation-derived epitopes within a single synthetic mRNA construct selected via bioinformatics algorithms based on HLA binding, expression, and immunogenicity. V940 showed interesting results in the adjuvant setting in a recent phase 2b clinical trial by Weber et al., comparing V940 plus pembrolizumab vs. pembrolizumab alone in resected melanoma. 157 patients were enrolled. The combination of mRNA-4157 and pembrolizumab led to longer recurrence-free survival (RFS) compared to pembrolizumab alone (18-month RFS 79% vs. 62%; hazard ratio (HR) 0.561; 95% confidence interval (CI) 0.309–1.017; *p* = 0.053), while maintaining a manageable safety profile [45]. The positive results of V940 encouraged the beginning of phase III, which is now ongoing (NCT05933577).

An innovative strategy could be using vaccines for targeting tumoral immune escape mechanisms. Kjeldsen et al. conducted a phase I and II clinical trial in 30 patients with metastatic melanoma, using the first-in-class, ‘off-the-shelf’ immune-modulatory peptide vaccine (IO102/IO103) against indoleamine 2,3-dioxygenase (IDO) and PD-L1 in combination with nivolumab. The rationale of this vaccine comes from the finding that, in the blood of patients with cancer (and some healthy patients), there are circulating cytotoxic T cells directed against IDO and PD-L1, which are expressed in melanoma cells but also in the tumor microenvironment (TME). Thus, the vaccine could stimulate T cells to kill cancer cells while inhibiting the production of immune-suppressive signals by the TME. The results from Cohort A (30 anti-PD1 therapy-naïve patients) and cohort B (10 anti-PD1 therapy-refractory patients) have been presented. For cohort A, ORR was 80%, which was higher (94%) among PD-L1-positive patients vs. 61.5% in PD-L1-negative patients. Interestingly, to assess whether the response rate was to be attributed to nivolumab or the vaccine, they compared the data with matched controls from the Danish Metastatic Melanoma Database (DAMMED) who received anti-PD1 monotherapy, showing that the ORR was significantly higher in the trial (79.3% vs. 41.7%). Notably, at a median follow-up of 45.3 months, mPFS was 25.5 months. For cohort B, the best overall response was stable disease (*n* = 2), the mPFS was 2.4 months, and the mOS was 16.7 months, suggesting that the vaccine in the ICI-refractory patients has limited activity [46].

### 3.2. Non-Small Lung Cancer (NSCLC)

Lung cancer is one of the most lethal neoplasms now, but immunotherapy has become a game-changer in prognostic and management; however, the tumor could develop mechanisms of resistance to those treatments, not providing long-term survival [47]. During the past decade, diverse studies have been developed to understand the role of vaccines in this kind of tumor to meet this need and provide long-lasting protection. Most of these studies were phase I and II trials, and many of them were prematurely finished, or disappointing results were achieved, and just some of them highlighted new pathways to follow future directions in investigating fields [48]. Those are shown below in Table 2.

The pivotal MAGRIT trial tested the intramuscular injections of recMAGE-A3 with AS15 immunostimulant or placebo during 27 months in patients with completely resected, MAGE-A3-positive NSCLC as an adjuvant treatment. RecMAGE-A3 is the is a recombinant form of the tumor antigen MAGE-A3, and the adjuvant AS15 included QS-21 (a saponin adjuvant), monophosphorylated lipid A, CpG 7909 (a TLR9 agonist) in a liposomal formulation. A third of all tested patients had a MAGE-A3-positive tumor. After a follow-up of 38.1 months, disease-free survival was not improved with the investigational vaccine (HR: 1.2; 95% CI 0.89–1.18; *p* = 0.74) [49].

TG4010 is a vaccine composed of a suspension of a recombinant modified attenuated and replication-deficient vaccinia virus strain Ankara (poxvirus) that codes for mucin-1 (MUC1). It was evaluated in a phase IIb trial in an untreated stage IV population. The study met its primary endpoint, demonstrating that >40% of patients were progression-free after 6 months period (43.2%), but no significant differences in overall survival were seen. The study showed, in an exploratory analysis of the subgroups, that the level of activated NK cells (CD16 + CD56 + CD69 + lymphocytes or TrPAL) had a better prognosis, suggesting that it could be a biomarker of good response to the vaccine [50,51,52]. Thus, in 2016, a phase IIb/III trial was carried out to study the role of this biomarker. The trial was randomized and compared chemotherapy plus placebo versus chemotherapy plus TG4010 vaccine in the first-line setting in a population of 222 patients. The primary endpoint was met, showing a better PFS in the population with higher values of TrPALs [53].

CIMAvax-EGF is a human recombinant EGF conjugated to a carrier protein that blocks the EGF-EFGR interaction, responsible for tumoral cell growth and proliferation. A phase III trial in 2016 with 405 patients was conducted comparing the use of CIMAvax-EGF vs. Best Supportive Care (BSC) after first-line treatment with four to six cycles of platinum-based chemotherapy. The trial did not show a statistically significant difference between groups in OS, but the trial demonstrated that CIMvax-EGF could increase the anti-EGF antibodies in the bloodstream, allowing them to inhibit the binding between EGF/EGFR [54]. A post hoc analysis showed the population with a higher concentration of anti-EGF had a higher survival rate, suggesting that anti-EGF titers had a probable positive correlation in survival [55,56].

Vx-001-201, a peptide vaccine that targets the universal tumor antigen telomerase reverse transcriptase, that could induce a cross-reactive immune response that may help to target tumor self-antigens that are not correctly identified by the immune system, was studied in a phase II trial as a maintenance treatment after showing in preclinical data that might show response in “cold” tumors, giving an alternative to patients with lower responses to immunotherapy. The trial shows promising results in low-immunogenic patients, resulting in a benefit in overall survival (20.7 months vs. 11.1 months, *p* = 0.011) [57].

The relevance of these results shown above should be taken cautiously since the majority of them have been developed in the pre-immunotherapy era in lung cancer treatment. The latest clinical trial with published results and the first study in the era of the ICIs was released in 2023. A phase III trial was conducted to study the role of OSE2101, a T-specific immunotherapy elaborated to promote the proliferation of cytotoxic T-lymphocytes against tumor-associated antigens that are commonly observed in NSCLC. The trial was designed to meet the need for an optimized treatment in the population known to be ICI-resistant (progression after initiating ICI treatment). Results suggested that secondary resistant population (progression after 12 weeks of treatment with ICIs) may benefit from OSE2101 with an improvement in OS in that population of months 11.1 vs. 7.5 months (HR 0.59, CI 95% 0.38–0.91, *p*= 0.017), improvement in post progression survival, quality-of-life (QoL), and time to worsening performance status (PS). More trials should be carried out to understand the role of OSE2101, but these promising results suggest that a pre-selected population may benefit from its use [58].

### 3.3. Pancreatic Adenocarcinoma

Metastatic pancreatic adenocarcinoma treatment is based on cytotoxic drugs, but its effectiveness is still scarce, with a high mortality rate. Novel therapies such as immunotherapy have not shown any benefit in overall survival or progression-free survival. This is mostly due to the characteristics of its TME, where the prominent desmoplastic reaction and the lack of significant T-cell infiltration, in addition to a low mutational burden, make pancreatic cancer an immunological “cold” tumor. Vaccines have lately emerged as a possible therapeutic approach, but most of the clinical trials remain in early stages [59,60,61]. In Table 3, we reported ongoing phase II and III trials with vaccine therapy in pancreatic cancer patients.

GM-CSF Transfected Pancreatic Tumor Vaccine (GVAX), a whole-tumor cell-based vaccine, was studied in the STELLAR phase II clinical trial in combination with CRS-207 (live, attenuated Listeria monocytogenes expressing mesothelin) and cyclophosphamide, with or without nivolumab in a previously treated metastatic population. Even though there were no changes in OS between arms, results from both arms were comparable to the OS seen in the NAPOLI trial that led to standard of care (SoC) in previously metastatic patients. Also, changes in the TME were more frequently seen in the vaccine plus anti-PD-1-treated group, observing an increase in CD8^+^ T-cell density and expression of PD-L1 in myeloid cells. These changes were associated with a better OS, suggesting that this population could benefit from combination immunotherapeutic therapies [62]. GVAX has demonstrated the ability to induce changes in TME, inducing a higher density of tertiary lymphoid aggregates (TLAs), showing that GVAX can produce immunomodulatory effects in the TME. These changes in pancreatic cancer TME by GVAX could lead to the development of a pathway for overcoming resistance to other immunotherapeutic agents [63], for which scarce positive results have been seen in pancreatic cancer. However, GVAX further studies have led to divergent results, especially in combination settings with immunotherapy [64,65,66]. Therefore, the results should be taken cautiously.

Peptide-based vaccines target prevalent tumor-associated antigens seen in pancreatic cancer, such as KRAS, carcinoembryonic antigen (CEA), Wilms’ Tumor Gene (WT1), or MUC1. At the beginning, few clinical trials were developed to target these antigens, predominantly phase I and in adjuvant settings [59,67]. These studies had some limitations, such as the small sample size or the lack of strong control. Also, the studies were powered to evaluate safety and to observe objective immune responses that could be translated into clinical responses. In addition, results of DFS and OS were mostly compared with previously published historical data [67,68,69,70,71,72]. These trials showed, in some cases, encouraging data that could lead to new design studies, like the OCV-CO1 vaccine, a peptide vaccine with epitope peptides derived from KIF20A, VEGFR1, and VEGFR2 in combination with gemcitabine in the adjuvant setting. Results showed, with its limitations, that OCV-CO1 was tolerable and safe, with a favorable median DFS of 15.8 months compared with other published data; the DFS was particularly more significant in the higher KIF20A expression subgroup population. Further studies are needed to conclude that expression of KIF20A could be a prognostic factor [67]. Also, the WT1 peptide–based vaccine, in combination with gemcitabine in advanced pancreatic adenocarcinoma, showed promising results in PFS improvement, especially in the metastatic population and in the population with a higher WT1-specific immune response. However, some limitations were seen, including small sample size, cross-over acceptance, and the use of gemcitabine alone as the comparison drug, since FOLFIRINOX and nab-paclitaxel in combination with gemcitabine have emerged as first-line settings [72].

The only phase III trial conducted with peptide vaccines and published results is the TeloVac trial, where the combination of chemotherapy with a telomerase-based vaccine (GV1001) was compared to chemotherapy alone in advanced pancreatic cancer. Not being able to meet its primary endpoint, not showing benefit in OS [73].

As seen, therapeutic cancer vaccines can stimulate significant immune responses, but these responses have not translated into clinical benefits [59] and have not met the expectations raised. But recent research and technological developments in mRNA technology are changing the field. A phase I clinical trial studied the effect of the vaccine Cevumeram after surgery in combination with atezolizumab versus mFOLFIRINOX in the adjuvant setting. Cevumeram consists of an individualized neoantigen vaccine based on uridine mRNA-lipoplex nanoparticles. The incorporation of uridine-modified mRNA aimed to overcome the limitations of mRNA stability, reduce innate immune sensing, and enhance translation in APCs. The trial evaluated the immune response in the patient by evaluating the induction of neo-antigen-specific T cells. 16 patients were treated with a follow-up of 18 months, and patients with an augmented immune response showed a longer recurrence-free survival (13.4 months, *p* = 0.003). These results, despite the small sample size, suggest that mRNA vaccines can induce immunity in historically known to be “cold” tumors with low responses to immunotherapy [74]. In an extended 3.2-year follow-up, the recurrence-free survival remained at 13.4 months, with the median not yet reached in the responder group [75]. New clinical trials are being developed using this thesis. IMCODE003 (NCT05968326), which will assess the efficacy and safety of the Cevumeram vaccine after surgery in a phase II clinical trial.

### 3.4. Breast Cancer

Breast cancer is the most frequently diagnosed malignancy in women worldwide and remains the leading cause of cancer-related mortality [76]. Treatment strategies vary based on molecular subtypes, with chemotherapy, endocrine therapy, and HER2-targeted agents forming the cornerstone of management. Despite significant therapeutic advances, metastatic disease and recurrence continue to pose major clinical challenges.

In recent years, immunotherapy has emerged as a promising avenue in breast cancer treatment. Although no vaccines have yet received regulatory approval, multiple clinical trials are underway.

Despite encouraging early-phase trial results, no breast cancer vaccine has demonstrated a significant therapeutic benefit in large-scale phase III studies. Most vaccine strategies rely on TAA-derived peptides [77], yet progress has been hindered by several challenges, including breast cancer heterogeneity and the need for tailored immunization approaches. Accordingly, vaccine development is now focused on distinct objectives: primary prevention and recurrence prevention.

Prophylactic breast cancer vaccines aim to prevent tumor development by targeting early premalignant lesions. Several studies are investigating potential precancerous targets, including α-lactalbumin, a breast-specific differentiation protein expressed during lactation and in approximately 55% of breast cancers, particularly in 75% of triple-negative breast cancers (TNBC), but not in normal breast tissue. Recent findings suggest that α-lactalbumin vaccination enhances T-cell infiltration and elicits strong antitumor immunity, effectively preventing breast cancer development [78,79].

Other preventive strategies are being explored for specific breast cancer subtypes [80]. In HER2-positive breast cancer, DC-based vaccines transfected with the recombinant adenovirus RGDAdVneu have been shown to induce HER2/neu-specific immunity [81], while peptide-based vaccines targeting the HER2 dimerization loop suppress tumor growth by inhibiting receptor phosphorylation and tumor proliferation in vivo [82].

Although none of these vaccines have yet received regulatory approval, several phase I clinical trials are currently assessing their safety and efficacy. For a comprehensive overview of preclinical breast cancer prevention vaccines, we recommend the work of Vinayak et al. [83].

Breast cancer vaccines designed to prevent recurrence are typically administered alongside adjuvant therapy to reduce the risk of relapse. Numerous TAAs are under investigation to develop safe and tumor-specific vaccines, with key targets including HER2, MUC1, etc. [84], with HER2 being the most extensively studied.

Among HER2-derived vaccines, E75 (nelipepimut-S, NeuVax) is the most extensively studied, having undergone Phase I–III clinical trials (NCT00841399, NCT00584789, NCT01479244. Comprising an HLA-A2/A3-restricted HER2 peptide combined with GM-CSF [85], E75 demonstrated good tolerability and induced an immune response, particularly in patients with low HER2 expression [86]. However, despite promising Phase II results, it failed to significantly improve DFS compared to placebo [87]. Nonetheless, meta-analyses [88] suggest a significant reduction in recurrence rates and improved DFS and OS in vaccinated patients. Moreover, E75 exhibited minimal toxicity, reinforcing its safety profile and potential for further clinical investigation.

AE37, unlike E75, which primarily stimulates CD8^+^ T cells for direct tumor cell lysis, is an HLA-unrestricted, MHC class II HER2-derived peptide vaccine that activates CD4^+^ helper T cells, enhancing immune recognition of HER2-expressing tumor cells. This mechanism not only promotes CD8^+^ cytotoxic T cell responses but also supports memory cell induction, which is critical for long-term immune surveillance and recurrence prevention. By facilitating antigen presentation, AE37 elicits a broader immune engagement, improving tumor cell recognition [89]. In Phase II clinical trials, AE37, administered with GM-CSF in HLA-A2^+^ breast cancer patients, demonstrated a favorable safety profile with minimal adverse effects. Subgroup analyses suggest potential clinical benefits, particularly in patients with advanced-stage disease, low HER2 expression, and triple-negative breast cancer. However, inconsistencies in efficacy outcomes highlight the need for improved patient stratification and further vaccine optimization [90].

GP-2, a HER2-derived peptide vaccine, is administered with GM-CSF (GLSI-100) to elicit an immune response against HER2/neu-expressing cancers. It has progressed through Phase I–III trials, with a Phase IB study in HLA-A2^+^/A3^+^ HER2^+^ patients confirming safety and a Phase II trial suggesting benefits in HER2-overexpressing patients [91]. Currently, the Phase III Flamingo-01 trial (NCT05232916) is evaluating GLSI-100 in breast cancer patients with residual disease or high-risk pathological complete response following neoadjuvant and adjuvant anti-HER2 therapy.

Beyond HER2, MUC1 has emerged as a promising target in breast cancer immunotherapy. In breast cancer, MUC1 is overexpressed in more than 90% of patients. Given its strong immunogenicity, MUC1 has been extensively studied as a vaccine target [92]. Various MUC1-based cancer vaccines, including subunit, DNA, viral vector, and dendritic cell vaccines, have been developed, often in combination with adjuvants such as BCG, QS-21, and GM-CSF to enhance immune responses [93].

Despite significant advancements, clinical outcomes remain variable, likely due to tumor-induced immune suppression and self-tolerance mechanisms. Further research is required to refine vaccine formulations and optimize immunogenicity, with the goal of improving the efficacy of MUC1-targeted breast cancer immunotherapy [93].

### 3.5. Prostate Carcinoma

Prostate neoplasms are the most prevalent malignancies affecting the male genitourinary system, high number of TAAs such as prostatic acid phosphatase (PAP) or PSA between others, makes it a suitable candidate for targeted vaccines even though, prostate cancer is historically known to be a “cold” tumor where immunotherapy has not met the expectations, and the results waited due to its intrinsic characteristics that confers an immunosuppressive tumor microenvironment. Hence, vaccines have emerged as a possible therapeutic approach in prostate cancer [94,95]. In Table 4, we reported some ongoing phase II and III trials with vaccine therapy in prostate cancer patients.

Sipuleucel-T is a cell-based vaccine obtained by incubating peripheral blood mononuclear cells with a prostate acid phosphatase-GM-CSF fusion protein. It is known to be the first FDA approved therapeutic vaccine in solid tumors due to the results from de IMPACT phase III trial involving 512 men with metastatic castration-resistant prostate cancer (mCRPC) asymptomatic or minimally symptomatic, where the use of Sipuleucel-T resulted in a 4.1-month improvement in overall survival (HR = 0.78, 95% CI, 0.61–0.98, *p* = 0.03) compared to placebo and an improvement in the rate of 3-year survival in the vaccine group (31.7%) with an acceptable safety profile. However, the therapy did not demonstrate a statistically significant improvement in PFS. In addition, it induced a significant increase in antibodies against antigen PA2024 with a positive correlation with OS [96]. In a posterior analysis, a new study was developed to explore the prognostic and predictive value of baseline variables in the IMPACT trial. The study suggested that patients’ PSA levels were the strongest independent baseline prognostic factor for response to Sipuleucel-T (*p* < 0.0001), suggesting that the greatest benefit was amongst patients with lower baseline PSA values and with other baseline prognostic factors (e.g., PS, LDH, etc.), providing a rationale for using this kind of therapy in earlier stages [97].

Other strategies have been studied to obtain the most optimal and synergic clinical outcomes, such as combination treatments. STAND, STAMP, and STRIDE trials [98,99,100] were three phase II trials developed to study the combination of Sipuleucel-T with anti-androgen receptor targeted therapy, whether concurrent or sequentially. At first, there was the thought that anti-androgen therapy could impact the immune function by inhibiting testosterone activity [99]. The trials, even with small samples, demonstrated that regardless of whether the agents were administered sequentially or concurrently, they did not affect the activation and induction of specific immune responses with well tolerated safety profile. In 2023, long-term survival results were published showing a median OS of 33 months [99].

More combination strategies have been studied in the mCPRC population, mostly phase II trials, such as Radium-223, radiotherapy, or other immunomodulatory agents like IL-7 or ICI [95,101,102,103,104]. Results were different depending on the combined treatment. Sipuleucel-T with IL-7 showed an enhanced immune response, with improved antigen-specific humoral and T cell proliferative responses [102], while Sipuleucel-T and radiotherapy [105] or radium-223 [101] did not result in an enhancement of the immune response. Finally, ICBs were studied, both ipilimumab [103] and atezolizumab [104], in phase II and phase Ib trials, respectively. The results showed disappointing outcomes with the ipilimumab study since no significant antigen-specific response was observed, with modest clinical outcomes. Atezolizumab, however, showed a favorable safety profile and may provide enhanced benefits, but further studies are needed to understand its relevance.

In addition, other kinds of vaccines (peptide vaccines, nucleic acid vaccines (DNA and mRNA), etc.) have been studied in many clinical trials. Results from these trials have been variable and mostly and sometimes inconclusive, leading to the interruption of the studies. The future has been focused on mRNA vaccines due to the technological advances in the analysis and synthesis of patient-specific tumor neoantigens [94].

### 3.6. Glioblastoma

Glioblastoma is considered another “cold tumor” where immunotherapy lacks activity due to the production of immunosuppressive cytokines, the less immunogenic produced neoantigens, and the high expression of PDL1 that leads to a down-regulation of T-lymphocyte proliferation and cytotoxic activity, producing an immunosuppressive TME. No changes have been observed in the past decades in its therapeutic approach. To overcome these difficulties, vaccines, as reported in Table 5, have emerged as a new therapeutic approach to improve clinical outcomes in this population [106].

Most clinical trials have been conducted on phase I and II peptide-based vaccines and dendritic cell-based vaccines, where these vaccines have shown some promising results on efficacy and safety (Table 6) [106]. Like the randomized, double-blind, placebo-controlled phase II clinical trial of ICT-107 (a dendritic cell vaccine targeting six antigens on both tumor and cancer stem cells) in patients with newly diagnosed glioblastoma after complete adjuvant treatment with concomitant chemo-radiotherapy. The study did not meet its primary endpoint, since mOS was not significantly improved. However, PFS showed a promising result with an improvement of 3 months versus the control group (11.2 months versus 9 months, *p* = 0.011) with an improvement in QoL parameters [107].

Personalized neoantigen vaccines have also risen as a potential therapy for these populations. This type of treatment has demonstrated in early clinical trials the ability to generate circulating neoantigen-specific immune-responses and an increase in the number of tumor-infiltrating T cells. A phase Ib trial was conducted to study the safety profile of ERC1671, in combination with adjuvant GM-CSF and preceded by a regimen of low-dose cyclophosphamide in patients who have progressed to first-line treatment [108]. Minimal toxicity was observed, and promising results in overall survival were seen, leading to the composition of a phase II trial, but in bevacizumab-naïve patients [109]. The interim data reported in August 2020 showed a highly significant improvement in OS benefit, showing a median OS of 10.5 months in comparison with historic controls that have reported a median OS of 5.3 months (log rank test, *p* < 0.0001). Therefore, the FDA recommended the early termination of this phase II trial, encouraging the start of a phase III trial due to its positive results observed [110].

A main limitation of phase I and II trials conducted in glioblastoma is the small number of enrolled patients, making it difficult to extract conclusions and hypotheses that could lead to the development of phase III trials. The reason for the lack of eligible patients could be the general poor performance status with rapidly progressive neurological symptoms or the use, in some cases, of high-dose corticosteroids. Also, the results observed are usually compared with historical controls, lacking a controlled arm in the trial. These reasons could explain why many phase II trials have failed to transition to phase III trials and why the results in phase III trials remain disappointing [106,107,108,111,112,113].

Rindopepimut, a peptide vaccine targeting the EGFR deletion mutation EGFRvIII, was studied in one of the largest trials, in combination with temozolomide for newly diagnosed EGFRvIII-expressing glioblastoma after surgery and/or chemoradiation therapy in a phase III randomized double-blind trial. 745 patients were enrolled, but the trial did not observe an improvement in median OS [114].

DCVax^®^-L, an autologous tumor lysate-pulsed dendritic cell vaccine, was evaluated versus SoC for newly diagnosed glioblastoma. 331 patients were enrolled in a randomized double-blind phase III trial. In the ITT population, mOS was 23.1 months, while in the subgroup of patients with methylated O-6-methylguanine-DNA methyltransferase (MGMT) was 34.7 months. This increase in subgroup population suggests a possible new therapeutic approach of combining temozolomide and DCVax-L [115]. Despite the positive results, the methodology of the trial was questioned; initially, PFS was the primary endpoint, but in the first published results, PFS was not reported. Finally, in a phase III prospective externally controlled cohort trial, OS was established as the primary endpoint; moreover, due to crossover, the placebo group was depleted, and OS was compared with historical reports. Here, the PFS was published, and it was not as expected: the median PFS was 6.2 months for the DCVax-L arm and 7.6 months for the placebo group (*p* = 0.47). However, the study met its primary endpoint, reporting a significant improvement in OS (19.3 months versus 16.5 months) [116].

## 4. Future Perspectives and Conclusions

Therapeutic cancer vaccines have achieved important milestones, yet their clinical efficacy in solid tumors remains modest, reflecting biological, clinical, and structural challenges that constrain their impact in routine oncology practice [117,118,119].

Tumor heterogeneity and the immunosuppressive tumor microenvironment (TME) present formidable barriers [117,118,119]. High genomic diversity complicates neoantigen identification, while tumors deploy multiple immune escape mechanisms, including regulatory T-cell recruitment, myeloid-derived suppressor cell accumulation, inhibitory cytokine secretion, and checkpoint molecule upregulation [117,119,120]. Vaccines tested in advanced disease states with high tumor burden have shown limited efficacy, whereas patients with lower tumor burden or earlier stage disease demonstrate more favorable immunologic responses, as exemplified by the KEYNOTE-942 trial showing sustained benefit of mRNA-4157 plus pembrolizumab in resected melanoma [119,121,122,123].

Personalized cancer vaccines require tumor sequencing, neoantigen identification, and GMP manufacturing, creating time-consuming, costly processes that limit accessibility and scalability [119,120]. Despite these hurdles, advances in mRNA and neoantigen-based platforms offer flexibility and rapid design capabilities [117,119,124]. Novel lipid nanoparticle formulations with improved lymph node targeting and dendritic cell-specific delivery enhance vaccine immunogenicity [125,126,127].

Integration of vaccines with immune checkpoint inhibitors (ICIs) holds substantial potential to synergistically overcome tumor-induced immunosuppression [117,119,127,128]. Clinical data demonstrate that vaccines prime intensified immunogenicity and expand neoantigen-specific T-cell repertoires, while ICIs prevent T cell exhaustion [127,128,129]. Combination approaches incorporating Treg depletion, metabolic modulators, and cytokine-based therapies show synergistic efficacy in preclinical models [130,131].

External factors also influence vaccine development. Public skepticism toward new vaccine technologies, amplified by the COVID-19 pandemic, may slow patient enrollment in trials, particularly for mRNA-based vaccines. Funding priorities, regulatory hurdles, and resource allocation remain additional considerations that could affect the pace of clinical translation [132].

Artificial intelligence (AI) and computational biology represent transformative avenues for cancer vaccine development [133,134,135]. AI-driven approaches integrate multi-omics data to enhance neoantigen prediction, incorporating peptide-MHC binding affinity, TCR recognition, antigen processing, and differential indices between mutant and wild-type sequences [132,133,134,136]. Machine learning models, particularly ensemble approaches, achieve positive predictive values exceeding 70–85% for top-ranked neoantigen candidates, potentially shortening development timelines and reducing costs [136,137,138].

As clinical data accumulate, standardized metrics for immunologic and clinical endpoints, coupled with a mechanistic understanding of vaccine-tumor interactions, will be essential [117,118,119]. Biomarker-guided approaches assessing tumor mutational burden, PD-L1 expression, immune infiltration, and neoantigen-specific T cell responses are critical for identifying patients most likely to benefit [118,119,139]. The convergence of advanced vaccine platforms, AI-driven neoantigen prediction, rational combination strategies, and biomarker-guided patient selection positions therapeutic cancer vaccines as a cornerstone of next-generation precision immuno-oncology [117,119,140].

## Figures and Tables

**Table 1:** Ongoing phase II and III trials with vaccine therapy in Melanoma patients.

NCT	Drug(s) Employed	Combination Treatment	Vaccines Target Antigen	Trial Phase	Trial Setting	Number of Patients	Current Status	Preliminary Results	Start Year
NCT05155254	Peptide-based Vaccine (IO102–IO103)	Immunotherapy	IDO/PD-L1	III	Stage III/IV unresectable	407	Active, not recruiting	N/A	2022
NCT05933577	mRNA-based individualized neoantigen Vaccine (V940)	Immunotherapy	Personalized targets	III	Adjuvant therapy for melanoma	1089	Active, not recruiting	N/A	2023
NCT04079166 (The SCOPE Study)	DNA plasmid-based vaccine (SCIB1)	Immunotherapy	TRP2/gp100	II	Stage III/IV unresectable melanoma	173	Recruiting	Improved the ORR to 85% without an increase in clinically meaningful adverse events.	2019
NCT04526899	mRNA-based Vaccine (BNT111)	Immunotherapy	NY-ESO-1, MAGE-A3, tyrosinase, and TPTE	II	Stage III/IV unresectable melanoma	184	Active, not recruiting	N/A	2021
NCT05269381	Personalized Neoantigen Peptide-Based (PNeoVCA)	Immunotherapy	Personalized targets	I/II	Stage III/IV unresectable melanoma	36	Recruiting	N/A	2022
NCT05912244	Peptide-based Vaccine (IO102–IO103)	Immunotherapy	IDO/PD-L1	II	Stage III/IV unresectable melanoma	43	Recruiting	N/A	2023

N/A: not available.

**Table 2:** Ongoing phase II and III trials with vaccine therapy in non-small lung cancer patients.

NCT	Drug(s) Employed	Combination Treatments	Vaccines Target Antigen	Trial Phase	Trial Setting	Number of Patients	Current Status	Preliminary Results	Start Year
NCT06751901	Personalized Neoantigen-based Peptide Vaccine	IO + RT	Personalized targets	II	Advanced NSCLC	10	Recruiting	N/A	2024
NCT06751849	Personalized tumor neoantigen- dendritic cell (DC) vaccine	IO + RT	Personalized targets	II	Advanced NSCLC	10	Recruiting	N/A	2024
NCT06472245 (ARTEMIA)	Peptide vaccine (OSE2101).	N/A	HER-2/neu, CEA, MAGE 2, MAGE 3, and p53	III	Advanced NSCLC	363	Recruiting	N/A	2024
NCT06015724 (DARANIVOVAX)	Peptide vaccine (Targovax TG-01/Stimulon QS-21)	Daratumumab + IO	KRAS	II	Advanced NSCLC	54	Recruiting	N/A	2024
NCT05950139	Peptide vaccine	ALK-inhibitors	ALK	I/II	Advanced ALK+ NSCLC	12	Recruiting	N/A	2024
NCT05557591 (EMPOWERVAX Lung 1)	mRNA vaccine BNT116 (FixVac Lung)	IO	TAAs	II	Advanced NSCLC	100	Recruiting	N/A	2024
NCT05344209 (LUNGVAC)	Peptide vaccine (UV1)	IO	hTERT	II	Advanced NSCLC	138	Recruiting	N/A	2022
NCT05269381	Personalized Neoantigen-based Peptide Vaccine (PNeoVCA)	CP + IO	Personalized targets	I/II	Advanced NSCLC	36	Recruiting	N/A	2022
NCT05242965	DNA vaccine (STEMVAC)	N/A	(CDH3, CD105, YB-1, MDM2 and SOX2	II	Advanced NSCLC	40	Recruiting	N/A	2023
NCT03970746 (PDC-LUNG-101)	Irradiated plasmacytoid dendritic cell vaccine	ChT + IO	NY-ESO-1, MAGE-A3, MAGE-A4, Multi-MAGE-A, MUC1, Survivin and Melan-A	II	Advanced NSCLC	73	Active, not recruiting	Promising ORR results in combination with pembrolizumab in first line setting	2019
NCT01720836	Peptide vaccine	N/A	MUC1	I/II	Localized and Locally Advanced NSCLC	30	Recruiting	N/A	2012

CP: cyclophosphamide. ChT: chemotherapy. IO: immunotherapy. N/A: not available. RT: radiotherapy.

**Table 3:** Ongoing phase II and III trials with vaccine therapy in pancreatic cancer patients.

NCT	Drug(s) Employed	Combination Treatments	Vaccines Target Antigen	Trial Phase	Trial Setting	Number of Patients	Current Status	Preliminary Results	Start Year
NCT06015724	Peptide vaccine (Targovax TG-01/Stimulon QS-21)	IO + Daratumumab	KRAS	II	Advanced Pancreatic Adenocarcinoma	54	Recruiting	N/A	2024
NCT05726864 (AMPLIFY-7P)	Peptide vaccine [Amph-CpG-7909]	N/A	KRAS	I/II	Advanced Pancreatic Adenocarcinoma	158	Active, not recruiting	N/A	2023
NCT05968326 (IMCODE003)	mRNA-based vaccine (Autogene cevumeran)	IO + ChT	Personalized targets	II	Advanced Pancreatic Adenocarcinoma	260	Recruiting	N/A	2023
NCT05964361	Interleukin-15-transpresenting Wilms’ tumor protein 1-targeting autologous dendritic cell (IL15-TransDC)	IL-15	WT1	I/II	Advanced Pancreatic Adenocarcinoma	10	Recruiting	N/A	2023
NCT05638698	Peptide-based vaccine (Tg01 Vaccine/Qs-21 Stimulon)	Balstilimab	RAS	II	Local or Locally advanced Pancreatic Adenocarcinoma	24	Active, not recruiting	N/A	2022
NCT04111172	Viral vector-based vaccine (Ad5.F35-hGCC-PADRE)	N/A	-	II	Local or Locally advanced Pancreatic Adenocarcinoma	81	Active, not recruiting	N/A	2020
NCT02451982	Tumor cell-based vaccine (GVAX)	CP + IO + Urelumab	-	II	Local or Locally advanced Pancreatic Adenocarcinoma	76	Recruiting	N/A	2016
NCT01088789	Tumor cell-based vaccine.	CP	-	II	Local or Locally advanced Pancreatic Adenocarcinoma	71	Active, not recruiting	N/A	2010

CP: cyclophosphamide. ChT: chemotherapy. IO: immunotherapy. N/A: not available.

**Table 4:** Ongoing phase II and III trials with vaccine therapy in breast cancer patients. mTNBC: Metastatic Triple Negative Breast Cancer. TNBC: Triple Negative Breast Cancer.

NCT	Drug(s) Employed	Combination Treatment	Vaccines Target Antigen	Trial Phase	Trial Setting	Number of Patients	Current Status	Preliminary Results	Start Year
NCT05329532 ModiFY study	Modi-1	Immunotherapy	Citrullinated peptides	I–II	mTNBC	114	Recruiting	Well tolerated Safety and efficacy data are supportive	2022
NCT05269381 PNeoVCA	Personalized neoantigen vaccine	Immunotherapy + Cyclophosphamide	Personalized targets	I–II	mTNBC	36	Recruiting	N/A	2022
NCT05455658	STEMVAC (DNA plasmid-based)	N/A	MDM2 YB1 SOX2 CDC25B CD105	II	Stage IB-III TNBC	33	Recruiting	N/A	2022
NCT06023277	ConvitVax	N/A	Autologous tumor cells + bacillus Calmette–Guérin (BCG) + formalin	1b/2	mBC	40	Not yet recruiting	N/A	2024
NCT05325632 (NATASHA)	HER-2 pulsed dendritic cell (DC1) vaccine	Trastuzumab + Pertuzumab + Paclitaxel	HER-2	2	Stage I–III, HER2+	53	Recruiting	Well tolerated improved radiologic tumor responses	2022

N/A: not available.

**Table 5:** Ongoing phase II and III trials with vaccine therapy in Prostate Cancer patients.

NCT	Drug(s) Employed	Combination Treatments	Vaccines Target Antigen	Trial Phase	Trial Setting	Number of Patients	Current Status	Preliminary Results	Start Year
NCT06636682	Tumor cell-based vaccine (FK-PC101)	Hormone therapy	Personalized targets	II	High-Risk Prostate Cancer Following Prostatectomy	100	Recruiting	N/A	2024
NCT06100705	Activated Autologous Dendritic Cells (Sipuleucel-T)	Testosterone Cypionate	PA2024	II	mCRPC	26	Recruiting	N/A	2023
NCT04989946	Plasmid DNA vaccine (pTVG-AR)	Hormone therapy + Immunotherapy	Androgen Receptor (AR)	I/II	Local High-Risk Prostate Cancer	60	Recruiting	N/A	2021
NCT04114825 (BRaVac)	Peptide-based vaccine (RV001V)	N/A	RhoC	II	Biochemical recurrence (BCR) within 3 years of radical prostatectomy (RP)	180	Active, not recruiting	N/A	2019
NCT04090528	Plasmid DNA vaccines (pTVG-AR) + (pTVG-HP)	IO	Androgen Receptor (AR) + PAP	II	mCRPC	60	Active, not recruiting	N/A	2019
NCT03600350	Plasmid DNA vaccines (pTVG-HP)	IO	PAP	II	non-metastatic, non-castrate prostate cancer (clinical stage D0/M0)	19	Active, not recruiting	Safe, immunologically active, prolonged time to disease progression, but did not eradicate the disease	2019
NCT02768363 (ULYSSES)	Viral vector-based cancer vaccines (CAN-2409)	valacyclovir	TAA	II	Intermediate-to-high-risk, localized prostate	187	Active, not recruiting	N/A	2016
NCT01436968 (PrTK03)	Viral vector-based cancer vaccines (ProstAtak)	Radiation therapy	TAA	III	Intermediate-high risk localized prostate cancer	711	Active, not recruiting	N/A	2011
NCT01197625	Autologous Dendritic Cells Loaded With mRNA From Primary Prostate Cancer	-	hTERT and Survivin	I/II	Local High-Risk Prostate Cancer after surgery	30	Active, not recruiting	N/A	2010

N/A: not available.

**Table 6:** Ongoing phase II and III trials with vaccine therapy in Glioblastoma Cancer patients.

NCT	Drug(s) Employed	Combination Treatments	Vaccines Target Antigen	Trial Phase	Trial Setting	Number of Patients	Current Status	Preliminary Results	Starting Year
NCT06805305	Autologous Dendritic Cell Vaccine (DOC1021)	pIFN + SoC	TAAs	II	GBM Adjuvant	135	Recruiting	N/A	2025
NCT06622434 (NAVIG-1)	Peptide-Based Vaccine	SoC	TERT and PTPRZ1	I/II	GBM Adjuvant	35	Recruiting	N/A	2024
NCT05685004	Personalized Neoantigen-based Peptide Vaccine	SoC	Personalized targets	II/III	GBM Adjuvant	120	Recruiting	N/A	2023
NCT05163080 (SURVIVE)	Peptide-based vaccine (SurVaxM)	SoC	Survivin	II	GBM Adjuvant	247	Active, not recruiting	N/A	2021
NCT04801147 (DENDR1)	Autologous Dendritic Cell Vaccine	SoC	TAAs	I/II	GBM Adjuvant	76	Active, not recruiting	N/A	2010
NCT04573140 (PNOC020)	mRNA vaccine NA-lipid Particle (RNA-LP)	SoC	TAAs	I/II	GBM Adjuvant	52	Recruiting	N/A	2021
NCT04523688	Autologous Dendritic Cells (DC) vaccine (Combi G-Vax)	SoC	TAAs	II	GBM Adjuvant	28	Recruiting	Safe and well-tolerated	2021
NCT03688178 (DERIVe)	Autologous Dendritic Cells (DC) vaccine (Human CMV pp65-LAMP mRNA-pulsed autologous DCs)	Varilimubab + Tetanus-diphtheria (Td) toxoid + SoC	TAAs	II	GBM Adjuvant	43	Active, not recruiting	N/A	2020
NCT03382977	VBI-1901 Enveloped Virus-Like Particles (eVLPs)	-	gB and pp65.	I/II	GBM Adjuvant	98	Recruiting	N/A	2017
NCT02649582 (ADDIT-GLIO)	(m)RNA-loaded dendritic cell (DC)	SoC	WT1	I/II	GBM Adjuvant	20	Active, not recruiting	N/A	2015
NCT02455557	Peptide-based vaccine (SurVaxM)	SoC	Survivin	II	GBM Adjuvant	66	Active, not recruiting	95.2% remained progression-free 6 months Median PFS was 11.4 months, median OS was 25.9 months	2015

N/A: not available; GBM: Glioblastoma.

## Data Availability

No new data were created or analyzed in this study. Data sharing is not applicable to this article.
